# Inherited Retinal Disease Therapies Targeting Precursor Messenger Ribonucleic Acid

**DOI:** 10.3390/vision1030022

**Published:** 2017-09-01

**Authors:** Di Huang, Sue Fletcher, Steve D. Wilton, Norman Palmer, Samuel McLenachan, David A. Mackey, Fred K. Chen

**Affiliations:** 1 Molecular Therapy Laboratory, Murdoch University, Murdoch 6150, Australia; 2 Centre for Ophthalmology and Visual Science (Incorporating Lions Eye Institute), The University of Western Australia, Nedlands 6009, Australia; 3 Perron Institute, 4th Floor A Block, Queen Elizabeth II Medical Centre, Verdun Street, Nedlands 6009, Australia; 4 Department of Ophthalmology, Royal Perth Hospital, Perth 6000, Australia

**Keywords:** alternative splicing, pre-mRNA splicing process, inherited retinal dystrophy, splicing correction, antisense oligonucleotides, retinitis pigmentosa

## Abstract

Inherited retinal diseases are an extremely diverse group of genetically and phenotypically heterogeneous conditions characterized by variable maturation of retinal development, impairment of photoreceptor cell function and gradual loss of photoreceptor cells and vision. Significant progress has been made over the last two decades in identifying the many genes implicated in inherited retinal diseases and developing novel therapies to address the underlying genetic defects. Approximately one-quarter of exonic mutations related to human inherited diseases are likely to induce aberrant splicing products, providing opportunities for the development of novel therapeutics that target splicing processes. The feasibility of antisense oligomer mediated splice intervention to treat inherited diseases has been demonstrated in vitro, in vivo and in clinical trials. In this review, we will discuss therapeutic approaches to treat inherited retinal disease, including strategies to correct splicing and modify exon selection at the level of pre-mRNA. The challenges of clinical translation of this class of emerging therapeutics will also be discussed.

## 1. Introduction

Inherited retinal diseases (IRDs) are a diverse group of clinically and genetically heterogeneous disorders. As a group, the prevalence varies for subcategories of IRDs, for example, approximately 1/4000 for non-syndromic retinitis pigmentosa [1] and 1/40,000 for cone rod dystrophies [2]. IRDs are characterized by bilateral progressive retinal degeneration which primarily affects the photoreceptor and the retinal pigment epithelium (RPE) cells but it may rarely affect other retinal cell types such as the Muller glia and bipolar cells. Some IRDs, such as Best disease and vitelliform macular dystrophy, can be restricted to the macular region (central retinal) whilst others, such as rod cone dystrophy (also known as retinitis pigmentosa) and choroideremia, affect cells distributed throughout the entire retina. IRDs can be inherited through X-linked, autosomal-recessive, autosomal-dominant or maternal uniparental (through mitochondrial DNA mutations) inheritance patterns or arise from de novo mutations of dominant genes [3,4,5]. Mutations in over 290 different genes are known to cause one or more subtypes of IRDs. Despite being the most common cause of blindness in the working age group, there are currently no proven treatments for IRDs [6].

There are several features that make the human eye an attractive target for innovative therapies. Firstly, the eye is a small and compartmentalized organ with a blood–retina barrier that limits diffusion of therapeutics out of the eye, minimizing systemic immune responses to biologics, vectors or transgenes. Treatments can be targeted to one eye, with the partner eye serving as a natural internal control. Moreover, a small dose of potential therapeutics can be injected at or near the target site, thereby achieving therapeutic drug concentrations locally and minimizing potential toxicity. The method of administration can be tailored, e.g., intravitreal injection to reach retinal cells in the internal retinal layers such as ganglion and Müller cells or subretinal injection to reach retinal cells in the external retinal layer such as photoreceptors and RPE. Optical transparency of the eye facilitates clinical evaluation of therapeutic effects by noninvasive techniques [7], such as high-resolution adaptive optics retinal photography, optical coherence tomography, electroretinography [8] and microperimetry. Through the transparent ocular media, laser photocogulation can also be readily applied to destroy treated retinal cells that display un-regulated growth. In the worst cases scenario, the eye, can be removed if local therapy does not control the serious unexpected adverse event from experimental ocular therapy. 

Neurodegeneration is a common endpoint in IRDs where primary cell apoptosis initiated by mutant gene expression is coupled with oxidative stress and leads to irreversible impairment in cellular function [9,10]. Currently, a number of innovative therapeutic approaches are under active consideration for the treatment of IRDs. These can be broadly divided into two categories, firstly, approaches that focus on neuro-replacement, and secondly, approaches that focus on neuro-preservation. The first of these is most appropriate for patients who present with end stage disease where few photoreceptors remain and retinal function is restored through cell replacement therapy or bionic vision via retinal visual prosthesis implant. Recent studies have discussed the benefits and disadvantages of these approaches in animal models and human patients with retinitis pigmentosa and age-related macular degeneration as their last resort for restoring vision [11,12,13,14,15]. In contrast, the ultimate aim of neuro-preservation strategies is to rescue neurons involved in neurodegeneration or slow down the degenerative process. Therefore, this second approach is most suitable for patients presenting earlier in the disease process before there is significant loss of retinal neurons. The goal of neuro-preservation is to restore the normal molecular products and intrinsic biological pathways that drive the progression of IRDs. This can be achieved using vectors or molecules designed to replace the gene (gene replacement therapy), edit the genome (gene editing) or alter precursor messenger RNA (pre-mRNA) processing and messenger RNA (mRNA) maturation. Further downstream, there are strategies that can allow translation to continue in the face of a nonsense or stop mutation, alter the incorrect peptide folding or restore the misdirection or mislocalization of the protein product of the mutated gene. There are also approaches to minimize the impact of impaired function of specific proteins or enzymes such as dietary modification to alter metabolites or the use molecules that mimic natural biological substrates to modify enzyme behavior or flux. At the post-translational modification level, there are also several targets for therapy including kinases, prenylation reactions, proteoglycan and proteolipid modifications and post translational cleavage. The desired clinical outcome from all in these approaches is the reduction in disease progression rate. Since approximately a quarter of exonic mutations causing inherited diseases in humans can affect splicing of pre-mRNA [16], we have chosen to focus on a review of the strategy of therapeutic modification of the mRNA splicing process. 

In recent years, several innovative therapeutic strategies that operate at the RNA level have become the focus of renewed attention, including RNA interference (RNAi), translational read-through of nonsense mutations and modifying pre-mRNA processing. RNAi silences gene expression, the mechanism of which involves the interaction of small interfering RNA (siRNA) molecules and endogenous multi-protein RNA-induced Silencing Complex (see Kole et al., for review [17]). A phase I clinical trial with siRNA-027, targeting vascular endothelial growth factor receptor-1, in patients with choroidal neovascularization caused by neovascular age-related macular degeneration has proved the safety of single-dose intravitreal injection, with stabilization or improvement in visual acuity and foveal thickness [18]. Although this provides an example of retinal gene expression manipulation, the target gene does not cause inherited retinal disease.

One mutation-specific therapeutic approach is the use of translational read-through inducing drugs (TRIDs). This class of molecules can restore full-length, functional protein by facilitating the recoding of a premature translational termination codon resulting from in-frame nonsense mutation. Since these types of mutations have been reported in many IRD associated genes, TRID treatments may have the potential to address a more substantial percentage of IRD cases. The application of TRIDs in various animal models of IRDs has achieved variable outcomes. Systemic delivery of aminoglycosides increases the number of surviving photoreceptor cells and preserves retinal function [19]. However, the limited efficacy of such drugs requires further investigation (see Nagel-Wolfrum et al., for review [20]).

## 2. The Pre-mRNA Splicing Process

**Figure 1:**
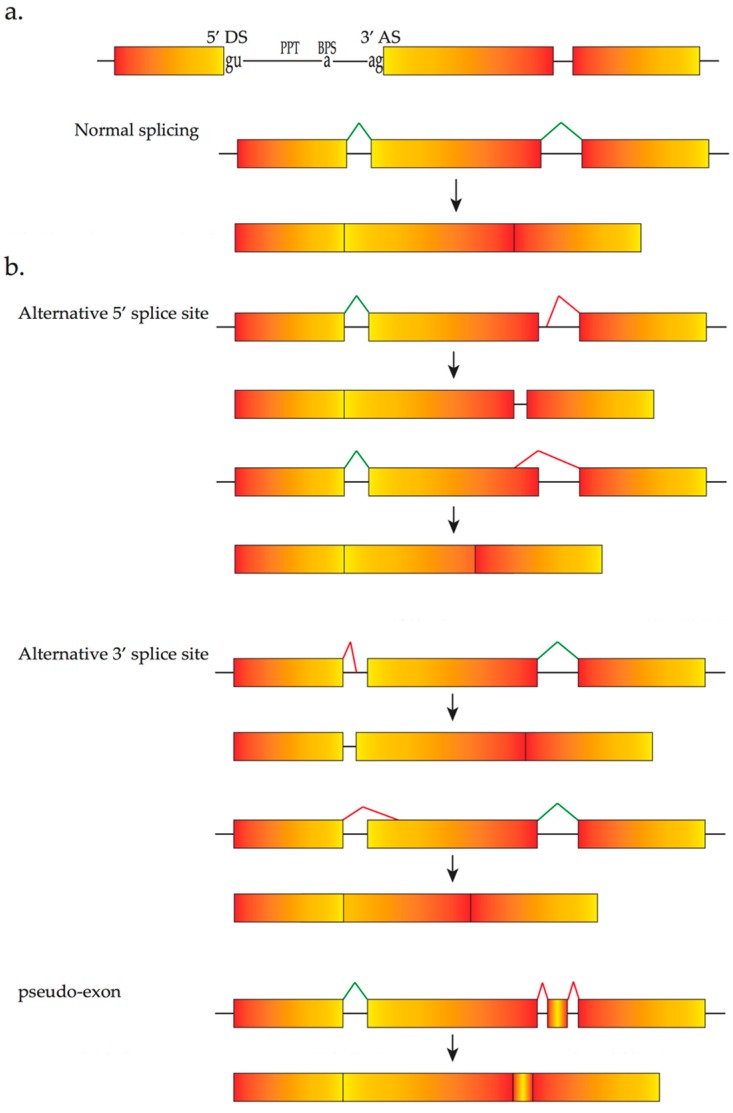
(**a**) Schematic representation of a *cis-*acting sequence, normal splicing and out of/in-frame transcripts from different mutations; (**b**) abnormal splicing resulting from various mutations of DNA that weaken/strenghthen the normal/cyrptic splice sites. Boxes represent exons and black lines are introns. Red lines, arrows and asterisks represent aberrant splicing processes and mutations, respectively.

The vast majority of human genes consist of protein-coding regions (exons) interspersed by non-coding intervening sequences (introns). After transcription, the gene transcript or pre-mRNA must be processed so that the non-coding sequences are removed whilst exonic regions are precisely joined together. Before being translated into protein, the gene transcript must undergo a series of complex constitutive processes to become a mature mRNA ready for translation in the cytoplasm. The correct recognition of exons and introns is regulated by *cis*-acting sequences that function as binding sites for *trans*-acting factors. The principal *cis*-acting sequences include the splice donor site at the 5′ end of the intron, the polypyrimidine tract, the branch point sequence and the splice acceptor site at the 3′ end of the intron, all of which are involved throughout the process of spliceosome assembly (Figure 1). The polypyrimidine tract, a variable number of pyrimidines (U’s and C’s), provides the substrate for recruitment of crucial factors and enhances recruitment of these factors to the branch point sequence at the same time. This process is indispensable to initial splicing. Both the splice donor and the splice acceptor sites are conserved regions that include the almost invariant sequences GU and AG, respectively. Other *cis*-acting sequences that are fundamental for mRNA splicing include intronic splicing enhancers and silencers that promote or suppress the recognition of nearby exons, exonic splicing enhancers and silencers that enhance or inhibit exonic recognition [21].

**Figure 2:**
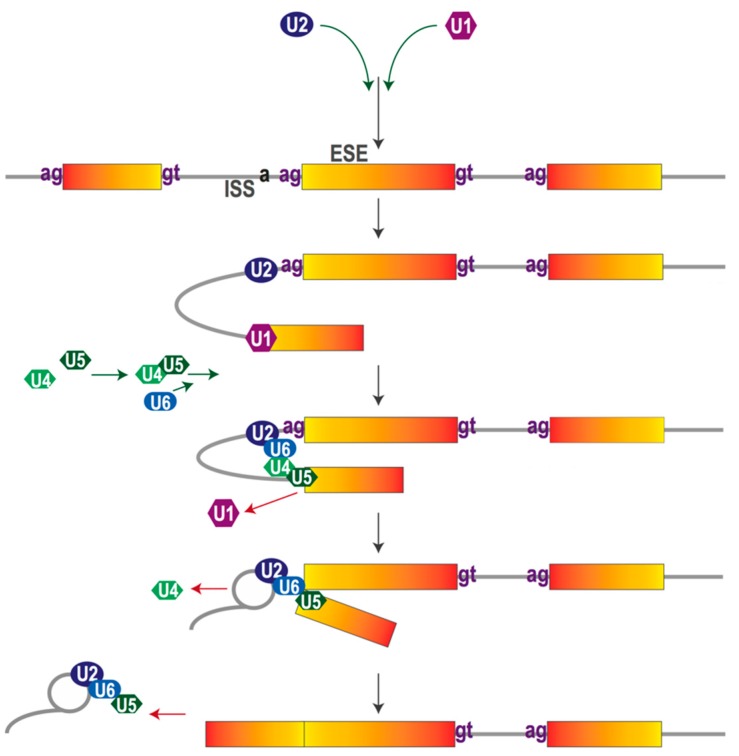
Small nuclear ribonucleo proteins (snRNPs) U1, U2, U4, U5 and U6 contain specific small nuclear RNA sequences of around 150 nucletorides in length which recognize and interact with *cis*-acting sequences guiding the splicing process [33].

Pre-mRNA splicing is catalyzed by the spliceosome, a macromolecular complex consisting of five uridine-rich small nuclear ribonucleic proteins (snRNP) U1, U2, U4, U5 and U6, each containing a small nuclear RNA (snRNA) combined with various proteins, as well as other less stably associated splicing factors [22]. These *trans*-acting factors are able to recognize and interact with specific pre-mRNA *cis*-acting sequences based on the base-pair principle. Examples of *trans*-acting factors are the heterogeneous nuclear ribonucleoproteins (hnRNPs) which mainly exert suppressive effects, and the serine- and arginine-rich (SR) proteins, which bind to intronic splicing enhancers or exonic splicing enhancers to promote splicing [23,24,25]. For more details of splicing regulatory factors/influences, readers are referred to other thorough reviews [26,27,28]). All these factors act either antagonistically or synergistically to determine the RNA sequence. When splicing begins, the U1 snRNP recognizes the splice donor site, followed by binding of the U2 snRNP to the branch point sequence. Subsequently, these snRNPs are combined with the U4/U5/U6 tri-snRNP complex, to create the mature spliceosome that catalyzes two trans-esterification reactions associated with a series of conformational changes. This process allows the joining of exons and the removal of the intervening introns [29] (Figure 2).

Natural variable exon usage or alternative splicing results from the use of alternative 5′ and 3′ splice sites, and is under tissue specific and/or developmental control. Exon skipping and intron retention are ubiquitous processes in higher eukaryotes that contribute to gene expression and proteomic diversity, thus representing a powerful evolutionary resource [30]. It has been estimated that over 80,000 protein-coding transcripts are encoded by fewer than 20,000 human genes and are translated into 250,000–1 million proteins [31]. Mutations in alternatively spliced exons of some disease-causing genes results in disorders such as X-linked retinitis pigmentosa [32]. One consequence of the complex sequence requirements for both constitutive and alternative splicing is the high incidence of mutations that cause abnormal splicing and thereby contribute in the genesis of human disease.

## 3. Inherited Retinal Diseases Due to Mutations That Affect Splicing and Spliceosome

Alternative splicing is highly regulated in a tissue-specific and development-specific manner, especially in the nervous system [34,35,36,37]. Tissue-specific splice isoforms contribute to diversity of gene expression, as well as contributing to the pathogenesis of a multiplicity of diseases. Goranka et al., found that the retina expresses the highest level of snRNAs, the key components in assembling snRNPs and U2-type spliceosome complexes, compared with other human tissues, indicating that the retina is highly dependent on splicing and subtle deficiencies in spliceosome components could lead to significant consequences [38]. The retina expresses the highest levels of spliceomal snRNAs [39], suggesting the retina has higher splicing requirements and a need for more delicate regulation than most other tissues. Murphy et al., demonstrated that photoreceptor cells have a distinct splicing pattern, that differs from central nervous system and inner retinal neurons. In photoreceptors, neuronal splicing factors are either downregulated or not expressed. According to these results, the unique splicing profile of photoreceptor cells may be regulated by a combination of mechanisms that assemble various splicing factors to form different exon recognition complexes based on RNA sequences [40]. Musashi 1, an RNA-binding protein that is indispensable for photoreceptor survival [41], is thought to be an important driver of the photoreceptor splicing profile and was shown to promote inclusion photoreceptor-specific exons, such as exon 2A of the *Ttc8* gene [40]. Further investigation is needed to identify other factors that may be involved in generating the photoreceptor-specific splicing profile. 

X-linked retinitis pigmentosa (XL-RP) is a subtype of retinitis pigmentosa (RP) that is clinically characterized by initial peripheral visual field constriction, night blindness and late-onset central vision loss associated with progressive retinal dysfunction [42]. The phenotype is caused by rod cell dysfunction and progressive photoreceptor cell death [43]. Approximately 60–80% of families with XL-RP are linked to mutations in the *retinitis pigmentosa GTPase regulator* (*RPGR*) gene [44]. The *RPGR* pre-mRNA undergoes alternative splicing to produce five major transcripts. In addition to mutations affecting the constitutively expressed transcript *RPGR* (exon 1–19), 55% occur in a glutamic acid-rich domain within the *RPGR*-open reading frame-15 (ORF15) region [45], indicating the significance of ORF15 in the occurrence of diseases. The exon 9a isoform, enriched in cone photoreceptors, contributes to approximately 4% of retinal transcripts. In one report, a single G to A nucleotide substitution (*RPGR* g.26652G > A) in an intronic splicing regulator was shown to upregulate the exon 9a-containing *RPGR* isoform [44]. Mutations in these tissue-specific exons or mutations that affect the splicing process of these regions result in primarily ocular diseases. 

**Figure 3:**
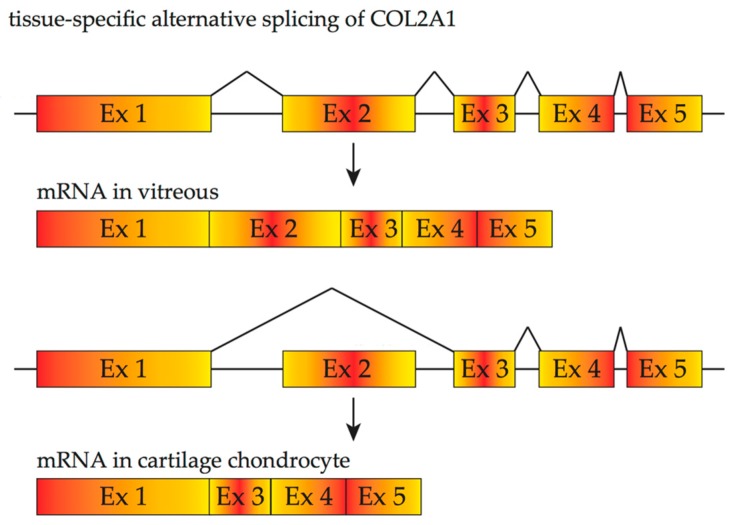
Tissue-specific alternative splicing of *COL2A1*. *COL2A1* mRNA containing exon 2 is primarily found in vitreous hyalocyte while mRNA lacking of exon 2 is found in cartilage chondrocyte.

*COL2A1* provides another example illustrating the significance of tissue-specific splicing variants in different diseases. Wagner’s disease has a purely ocular phenotype, mainly associated with vitreous degeneration and/or retinal pigmentation beginning in adolescence, whereas Stickler syndrome type I, an autosomal dominant disease, shows extra-ocular manifestations as well as vitreous anomaly and progressive ocular complications such as retinal detachment. Either of these disorders can be caused by mutations in the *COL2A1* gene. Two forms of procollagen II are produced from *COL2A1* as the result of alternative splicing of exon 2 (Figure 3). A longer form containing exon 2 is predominantly expressed in the vitreous body, whilst a shorter form lacking exon 2 is mainly found in adult cartilage [46,47]. Such features of alternative splicing explain the variability of phenotypes associated with *COL2A1* mutations: mutations in a tissue-specific alternatively spliced exon of *COL2A1* may result in Wagner’s disease or ocular only Stickler syndrome whilst Stickler syndrome due to mutations of *COL2A1* without involving exon 2 affects both cartilage and vitreous. For instance, Cys86Stop [48], caused by a C to A nonsense mutation, or Cys57Stop, caused by a frame-shift mutation are located in *COL2A1* exon 2 [44], and these mutations result in few or no extra-ocular manifestations [49]. 

Some genes that undergo alternative splicing can cause syndromic retinal degeneration, as well as isolated retinal dystrophy. For example, an exonic splice-site mutation in of one of the causative genes of Bardet-Biedl syndrome, *BBS8*, has been reported to cause nonsyndromic recessive RP [50]. In this report, homozygous splice-site mutation of *BBS8* resulted in the skipping of 30 bp of exon 2a sequence, an alternatively spliced exon expressed exclusively in the retina and primarily in photoreceptor cells. Mutation-induced missplicing of exon 2a of *BBS8* was later shown to produce a frameshift mutation in photoreceptors, but not in other cell types that normally exclude this exon [51]. Other genes that undergo alternative splicing, such as *MYO7A* [52], *CDH23* [53], *USH1C* [54], also play an important role in isolated retinal dystrophy or syndromic disease. 

According to a recent report, the two major mutation patterns in genes causing retinal diseases are missense and nonsense mutations that account for 34% and 15% of retinal diseases, respectively [21]. It is important to note the overlap between protein-coding sequence and *cis*-acting elements that participate in the splicing process [16]. Therefore, missense and nonsense variations may not only change protein structure and function, but also alter the splicing mode [55,56], It has been estimated that 25% of missense or nonsense pathogenic mutations induce abnormal splicing, exon skipping or activation of cryptic splice sites (Figure 1b) [16]. This proportion will increase by including mutations that affect crucial intronic splicing sequences.

**Figure 4:**
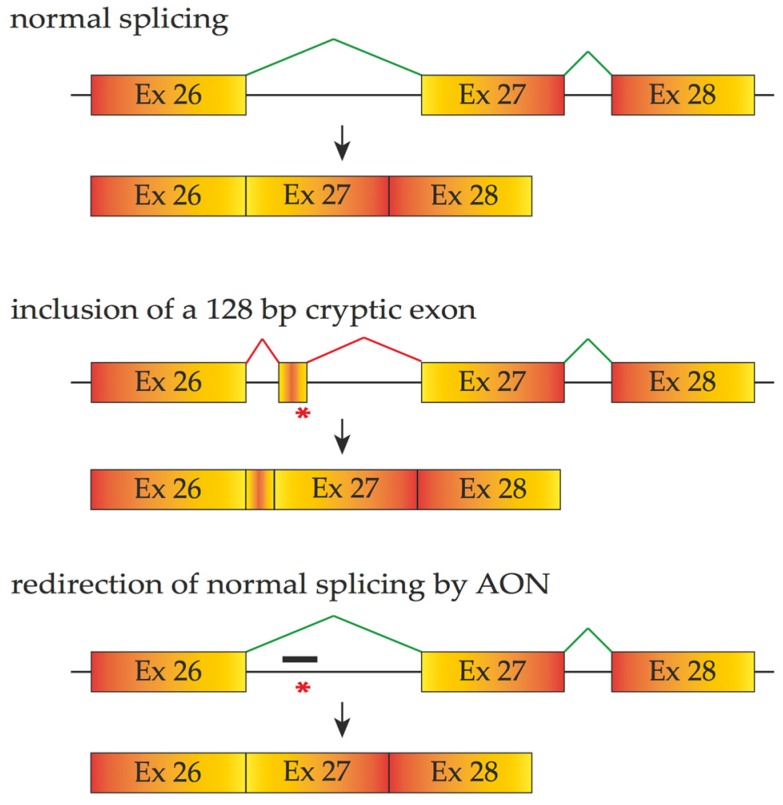
The schematic demonstrates how an intronic mutation (asterisk) that creates a donor splice site results in the inclusion of a 128 cryptic exon in the *CEP290* mRNA. Antisense oligonucleotides designed to mask this cryptic splice site (thick black line) prevent recognition of the cryptic exon by the splicing machinery and restores normal splicing products. Boxes represent exons and lines represent introns, red lines and asterisks represent aberrant splicing and mutation.

Small deletions and insertions account for approximately 23% of retinal diseases and can lead to premature termination codons and altered splicing outcomes through reading-frame disruption. The mRNAs carrying premature termination codons are eliminated through a nonsense-mediated decay mechanism. However, some premature termination codons may disrupt exonic splicing enhancers, resulting in the exclusion of the affected exon from the final mRNA [57]. Gross DNA deletions or rearrangements are less common, accounting for 12% of retinal dysfunctions. Mutations residing within non-coding introns could change the amino acid sequence of a protein by affecting splicing patterns. For instance, among the various mutations causing Leber congenital amaurosis (viz. LCA and Online Mendelian Inheritance in Man Entry 204000), a deep-intronic point mutation (c.2991 + 1655A > G) of *CEP290* is one of the most frequent mutations that act by introducing a cryptic splice donor site, thus resulting in inclusion of a 128 bp pseudo-exon encoding a premature stop codon (Figure 4) [58].

In addition to mutations that affect the splicing of specific retinal genes, mutations in genes associated with ubiquitously core snRNP proteins, such as PRPF3, PRPF4, PRPF6, PRPF8, PRPF31, SNRNP200/BRR2 and splicing factors such as RP9 and DHX38 lead to autosomal dominant retinitis pigmentosa [59].

## 4. Therapeutic Induced Alternative Splicing

### 4.1. Antisense Oligonucleotides

Antisense oligonucleotides (AONs) are chemically synthesized to target their complementary pre-mRNA. These 8–50 nucleotide, single-stranded molecules can be precisely targeted based on the Watson–Crick base-pairing principle. AONs can modulate splicing by interfering with recognition of splice sites, a process that leads to the generation of alternatively spliced target transcripts [60]. The application of AONs that inhibit viral replication in vitro was first reported in Rous sarcoma cells by Zamecnik and Stephenson in 1978 [61], who also showed that chemical modifications at the 5′ and 3′ ends of the oligonucleotide reduce degradation by cellular nucleases and thus increase stability. Since the 1970s, much research has focused on two basic chemistry modifications (backbone and sugar modification) of AONs with the goal of optimizing activity, binding strength and specificity [62].

AON therapy was first applied to modulate gene expression by inducing enzymatic degradation of the target mRNA and removing disease-causing gene products by endogenous ribonuclease H1 (RNase H1)-mediated cleavage. Research has shown that RNase H1 is the only nuclease that participates in activities of DNA-like AONs and the RNA cleavage happens only when a DNA-RNA heteroduplex is formed [63]. Crooke found that RNase H1 participates in various RNA metabolic activities with P32, a binding partner that enhances the specificity of this cleavage. Fomivirsen (ISIS 2922), a 21-nucleotide sequence complementary to the mRNA of the immediate early region 2 of human cytomegalovirus has been applied to clinical trials to decrease the viral load in Acquired Immune Deficiency Syndrome patients with viral retinitis [64]. The early research has found a correlation between the inhibition of immediate early protein expression and reduction of mRNA that is consistent with RNase H effects [65]. 

The rapid degradation of early AONs by endo- and exonucleases was a substantial limitation and led to the development of alternative AON chemistries. To improve AON performance, several different chemistries have been applied to their design including the use of the phosphorothioate internucleotide (PS) backbone. In this modification, one of the non-bridging oxygen atoms in the backbone is replaced with a sulphur atom, creating a linkage that is more resistant to degradation and still retains a negative charge that improves its cellular uptake [66,67] while retaining the ability to activate RNase H [68,69]. Further modification at the 2’ position of the ribose sugar led to the generation of AONs with a PS backbone but lacking RNase H induction. The 2’-O-methyl (2’OMe) and 2’-O-methoxy-ethyl (2’-O-MOE) AONs are two widely used AONs, with structural properties that dramatically increase oligonucleotide resistance to degradation, promote protein-binding strength and avoid inducing RNase H activity [17,70]. Moreover, the sequence-independent toxicity associated with immune stimulation is generally avoided [69]. Other chemical modifications of this type include locked nucleic acid (LNA) and 2’-O, 4’-C-ethylene-bridged acids (ENA). Peptide nucleic acid (PNA) and phosphorodiamidate morpholino oligomers (PMOs) are more recent modifications (Table 1).

Both LNA and PNA not only have strong affinity for target RNA but also are unable to support RNase H activity. PMO compounds are resistant to nucleases and proteases, and do not support RNase H. This modification transforms the molecule into an uncharged compound that resists further degradation. For more comprehensive information on the diverse chemistries for AONs, readers are referred to more specific reviews [68,71,72].

The advent of these novel AON chemistries has provided new possibilities for therapeutic modulation of gene expression. RNase H activity is not required, indeed would be counter-productive, when the therapeutic goal is to intervene in the splicing process. Antisense compounds can modulate the splicing process, by blocking access of cellular machinery to target pre-mRNA and mRNA thus leading to outcomes such as exon skipping, translation suppressing, RNA folding and mRNA degradation by the tRNA-processing ribozyme. Such compounds can be dressed with more extensive chemical modifications because they do not need to exploit cellular enzymes for their activities [17].

Recently, there has been growing interest in AONs because of the success of ongoing clinical trials. Splice-switching oligomers that treat Duchenne muscular dystrophy (DMD) and spinal muscular atrophy (SMA) have reached phase III trials [73,74,75,76], and of particular note, the US Food and Drug Administration has recently granted accelerated approval to Exondys51 for the treatment of a major subgroup of DMD. Nusinersen, another therapeutic drug that exploits the antisense mechanism has been approved in USA for intrathecal use in patients with SMA [77].

In ophthalmology, the use of a 2’OMe-modified AON on a PS backbone to cover the cryptic spice site created from c.2991 + 1655A > G of *CEP290* mutation in fibroblasts from LCA patients, thus redirecting the splicing process, was shown to be effective and caused a three to 4.5-fold upregulation of wild-type mRNA in LCA patient-derived fibroblasts equaling that in wild-type cells [82]. Other lab research and animal experiments using the same type of AON to exclude the same intron retention have revealed that the delivery of either naked AON or adeno-associated virus (AAV)-packaged AON increased normal *CEP290* splicing products and reduced aberrant splicing outputs of *CEP290* (c.2991 + 1655A > G)*.* This observation indicates that both AON delivery methods are promising candidates for splice correction therapy for *CEP290*-associated LCA [83,84]. Other AONs are under clinical investigation for treatment of inherited ocular disease as well. A significant advantage of AONs designed to downregulate growth factors (crucial to form ocular neovascularization) in diabetic retinopathy has been observed by Hnik et al. [85].

### 4.2. Engineered Small Nuclear Ribonucleic Acid (snRNA)

As mentioned in Section 2, snRNPs are a group of crucial *trans*-splicing factors that bind to complementary *cis*-acting sequences and trigger the splicing process. U1-based therapies target the first step of splicing since exons in the precursor mRNA are first recognized through the base-pairing between U1 snRNA and the splice donor site sequence.

Changes introduced into the U1 snRNA sequences to increase complementarity to the mutated splice site sequence have been used to strengthen the binding and improve recognition of the mutated splice donor sites, and restore correct splicing products. This approach contributes to correction of endogenously expressed transcripts and increases full-length products whilst reducing the proportion of aberrant protein. For example, Glaus et al., has investigated engineered U1 snRNA isoforms to correct exon 10 skipping of *RPGR* due to c.1245 + 3A > T, a novel splice donor site mutation in X-chromosomal RP patient-derived fibroblasts [43]. Similarly, Tanner et al., showed that a novel splice site mutation at the last nucleotide of exon 4 in rhodopsin (*RHO* c.936G > A) results in missplicing. Mutation-induced splicing could be corrected using a mutation-adapted, engineered U1 snRNA in COS 7 cells as well as in mouse retinal explant cultures. According to this study, the effect of mutation-adapted U1 snRNA relates to the strength of the donor site that is affected by certain mutations [86].

The efficacy of mutation-adapted U1 snRNA has also been demonstrated by Schmid et al., in Bardet-Biedl syndrome patient-derived cells [87]. In their study, a dose-dependent therapeutic effect was observed in both minigene assays and endogenous transcripts when mutation-adapted U1 snRNA was used to correct aberrant splicing products resulting from c.479G > A, a donor site mutation in exon 5 of *BBS1.* [87]. The combined treatment of mutation-adapted U1 snRNA and U6 snRNA that maintains exon recognition after U1 dissociation has also been shown to be capable of decreasing both exon skipping and intron retention due to a mutation occurring at +5 of the donor splice site [88]. 

Antisense sequences incorporated into a snRNP particle against junctions of specific exons can be delivered to cells by using viral vectors, therefore being protected from degradation as well as being transcribed in the nucleus. Goyenvalle et al., reported that AONs inserted into the lentiviral vector-mediated modified-U7 snRNA constructs induced specific exon skipping in myoblasts from DMD patients. Moreover, multiple U7 snRNAs delivered by a single AAV vector resulted in multiexon-skipping in transgenic mice carrying the entire human DMD locus in vivo [89]. Different U7 snRNAs are used to shuttle various effective antisense sequences to different key splicing elements that they are designed to target. This emerging area for therapeutic development of alternative splicing approaches in non-ocular diseases brings hope for its future application in ocular diseases including inherited retinal disease. 

### 4.3. Splicesome Protein Modulators

Modulation of splicing can also be achieved through small molecules that bind or inhibit specific spliceosome components or their kinases.

Additionally, PTK-SMA1, a tetracycline derivative with a modification of the tetracycline backbone at the C7 position, has been shown to promote survival of motor neuron 2 (*SMN2*) exon 7 splicing, probably by binding to its *SMN2* pre-mRNA directly or through one of the splicing machinery components or a related protein that participates in splicing [90]. SMN splicing modifier SMN-C1 has also been shown to be effective in promoting the inclusion of exon 7 and production of SMN protein in both human cells and transgenic SMA mouse models [91]. It has been shown that the spliceosome component, SF3b can be interfered by spliceostatin A [92], an inhibitor that prevents SF3b binding to U2 snRNP at the branch point adenosine thus preventing splicing. 

As mentioned above, the SR-rich protein is one of the most prominent mediators of splice site recognition. The SR protein interacts with pre-mRNA at its unique RS domain, which includes an arginine/serine-rich domain and N-terminal RNA recognition motif, sites that can be highly phosphorylated on their serine residues. This phosphorylation has a positive effect on SR protein activity, and several SR kinases have been discovered, including SRPK1 [93]. Interestingly, the positive effects of SR protein association with *cis-*acting sequences appear to be position-dependent. SR promotes splice site selection by binding to ESEs and suppresses the selection by binding to its intronic corresponding sites [94]. Recently, extensive application of SR protein and SR protein kinases has revealed its importance in regulating alternative splicing to vascular endothelial growth factor for instance, and to human diseases such as cancers [23,95]. For further information, readers are referred to other reviews [25,29]. 

### 4.4. Pre-Trans-Splicing Molecules

**Figure 5:**
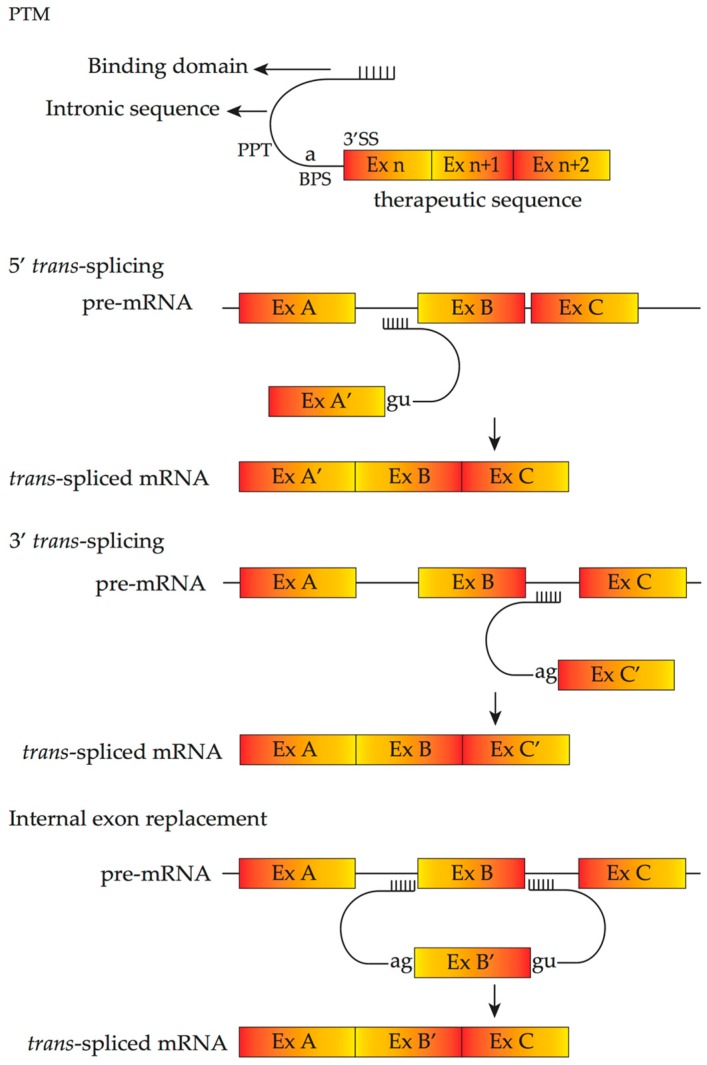
Schematic demonstrates **pre-*trans-*splicing molecule** (PTM) design and *trans*-splicing mechanism of three kinds of spliceosome-mediated RNA *trans*-splicing (SMaRT). The PTM includes a binding domain, an intronic sequence containing the polypyrimidine tract (PPT), the branch point sequence (BPS), the splice acceptor site (SS), and the therapeutic sequence. PTMs can be designed to replace 5’-exons, 3’-exons or internal exons [97]. The binding domain recognizes the target intron on endogeneous pre-mRNA by base pairing, thus, for **5′-*trans*-splicing**, resulting in a trans-spliced mRNA in which 5’ exons are replaced with the therapeutic sequence and for **3′-*trans*-splicing**, resulting in a trans-spliced mRNA in which 3’ exons are replaced with the therapeutic sequence. For **internal exon replacement**, binding domains are included at both ends of the PTM, resulting in a trans-spliced mRNA in which targeted internal exons are replaced with the therapeutic sequence.

Spliceosome-mediated RNA *trans*-splicing (SMaRT) is another mRNA repair strategy. *Trans*-splicing is a natural splicing phenomenon occurring at much lower frequencies compared to *cis*-splicing processes [96]. *Trans*-splicing can generate a new recombination of mRNA that contains the 5′ part of one pre-mRNA and the 3′ part of another pre-mRNA. SMaRT utilizes a pre-*trans-*splicing molecule (PTM), which contains a therapeutic replacement sequence to be introduced to the target mRNA, an antisense binding domain that anneals with specific intronic sequences of target pre-mRNA and an artificial intron sequence that contains all essential elements for splicing. Depending on the orientation of PTM, three kinds of SMaRT are available; 5′- or 3′- *trans*-splicing and internal exon replacement, which replace the 5′-exons, 3′-exons and internal exons, respectively (Figure 5).

There are five main benefits of this method. Firstly, it is mutation-independent and thus the same pre-*trans-*splicing molecule can be used for various mutations in the same region of the transcript. Secondly, it occurs only when and where the target pre-mRNA exists, thereby preserving the expression levels and tissue specificity of the repaired transcripts [98]. Thirdly, endogenous control of the splicing process is preserved. Fourthly, since only part of the gene sequence needs to be replaced, the SMaRT approach requires a shorter therapeutic coding sequence than gene therapy approaches that aim to replace the entire gene. Finally, such *trans*-splicing molecules make it possible to increase wild-type protein and decrease mutant protein simultaneously. Recently, a study by Adeline et al., demonstrated that by targeting intron 1 of *RHO* pre-mRNA and thereby replacing exon 2 to exon 5, the level of *trans*-splicing can be high enough to achieve desired therapeutic effects in patients with retinitis pigmentosa, a genetic disease with dominant inheritance [96]. Several factors that can increase the efficiency of PTM and promote *trans*-splicing have been identified, including the location of the binding domain [99]; modification of therapeutic replacement sequences by inserting splicing-promoting sequences; modification of the length of the binding domain; introduction of an intronic splicing enhancer; the PTM binding the intron 5′ part; and the ratio between PTM and its target pre-mRNA [96]. The PTM must enter the nucleus to be efficiently utilized before endogeneous *cis*-splicing can occur. Also, the in vivo study of *trans*-splicing by using a humanized mouse model of RP due to *RHO* mutation revealed that up to 40% of the mRNA can be repaired. This study has paved way for the application of SMaRT technology in the treatment of dominant IRDs.

## 5. Delivery of Therapeutics Inducing Alternative Splicing

Since IRDs in most cases involve in ocular posterior segment, two main intraocular delivery systems are taken into consideration: intravitreal injection and subretinal injection. Targeting drug delivery to the eye allows therapeutic drug concentrations to be achieved rapidly and locally, with smaller amounts required compared with systematic administration.

Intravitreal injection has been shown to be a promising route of administration with several advantages. It is more tolerable to patients and easier for ophthalmologists to perform. Though there are no current clinical trials investigating AONs to treat IRDs, some studies have applied AONs to ocular diseases in animal models. Gerard et al., successfully altered *CEP290* and *ABCA4* splicing in retinal cells of a wild-type mouse by intravitreal injection of 2’OMe phosphorothioate AONs. Both oligos and altered *CEP290* mRNA can be detected one month later after a single injection [100]. No adverse effect was observed during the one month follow up in their study. Murray et al., demonstrated that, in a rat model with autosomal dominant RP, AONs that were delivered to inhibit *RHO* allele containing P23H mutation can be measurable up to 60 days after a single intravitreal injection [101]. Similarly, side effects such as inflammation or toxicities were not observed in this study. 

Compared to intravitreal delivery methods, subretinal administration show some limitations. Only part of the retina can be targeted (20–60% depending on the operation) [84] and the procedure carries the potential risk of rhegmatogenous retinal detachment. Garanto et al., explored the therapeutic potential of the naked AONs (intravitreous injection) and AONs sequence subcloned into a modified U7 snRNA gene delivered by adeno-associated virus (subretinal injection) in humanized mouse models that carry intronic mutation in CEP290 (c.2991 + 1655A > G). Both approaches resulted in a dramatic decrease of aberrant *CEP290* transcripts up to one month after injection. No negative effect such as degeneration of photoreceptor cells and compromised retinal structure were observed [84]. The feasibility of *trans*-splicing has been further demonstrated by Berger et al. [96], who tested the most effective PTM by subretinal injection of AAV vectors into mouse models of RP. For further information on administration methods, readers are referred to other reviews [102,103].

## 6. Future Directions

Splicing intervention approaches targeting pre-mRNA have become one of the emerging therapeutics in modern medicine after its successful use in DMD and several AON-based drugs such as Exondys51 and Nusinersen (for SMA) have been approved [61]. Although AON-based therapies have potential to improve retinal function or slow down the rate of retinal degeneration in ocular disease, the heterogeneity of IRDs pose great challenges. To evaluate the therapeutic effect of numerous AONs designed individually requires a great deal of time and resources. The currently ongoing preclinical trials using AONs to treat IRDs are at a very early stage and longer follow-up will be needed to assess safety and efficacy. Efforts to safely and effectively deliver AONs to the retina, increase cellular uptake in vivo, limit off-target effects [72], define the optimal treatment window and examine potential toxicity and immunostimulatory effects [104] reflect the many issues still to be explored. Further research is required to obtain a better understanding of the complex interactions between small molecules (e.g., snRNP, SR protein and hnRNP), functionality of various splicing regulatory elements to aid in a more detailed and mature prediction of specific transcripts and strength of splice sites.

## Figures and Tables

**Table 1:** Examples of chemical modification in antisense oligonucleotides.

AON Modifications	Chemical Structure	Support RNase H	Refs.
Phosphate backbone	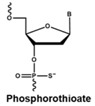	N/A	[78]
Sugar modification	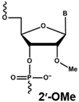	No	[79]
	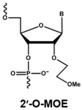	No	[79]
	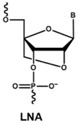	No	[80,81]
Non-ribose modification	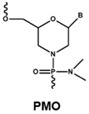	No	[78]
	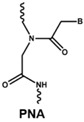	No	[79]
